# Intestinal Dysbiosis and the Developing Lung: The Role of Toll-Like Receptor 4 in the Gut-Lung Axis

**DOI:** 10.3389/fimmu.2020.00357

**Published:** 2020-03-05

**Authors:** Stephen Wedgwood, Kimberly Gerard, Katrina Halloran, Ashley Hanhauser, Sveva Monacelli, Cris Warford, Phung N. Thai, Nipavan Chiamvimonvat, Satyan Lakshminrusimha, Robin H. Steinhorn, Mark A. Underwood

**Affiliations:** ^1^Department of Pediatrics, UC Davis School of Medicine, Sacramento, CA, United States; ^2^Division of Cardiovascular Medicine, Department of Internal Medicine, UC Davis Health System, Sacramento, CA, United States; ^3^Department of Veterans Affairs, Northern California Health Care System, Mather, CA, United States; ^4^Department of Hospital Medicine, Children’s National Health System, Washington, DC, United States

**Keywords:** intestinal dysbiosis, TLR4, pulmonary hypertension, bronchopulmonary dysplasia, premature infant, Enterobacteriaceae

## Abstract

**Background:**

In extremely premature infants, postnatal growth restriction (PNGR) is common and increases the risk of developing bronchopulmonary dysplasia (BPD) and pulmonary hypertension (PH). Mechanisms by which poor nutrition impacts lung development are unknown, but alterations in the gut microbiota appear to play a role. In a rodent model, PNGR plus hyperoxia causes BPD and PH and increases intestinal Enterobacteriaceae, Gram-negative organisms that stimulate Toll-like receptor 4 (TLR4). We hypothesized that intestinal dysbiosis activates intestinal TLR4 triggering systemic inflammation which impacts lung development.

**Methods:**

Rat pups were assigned to litters of 17 (PNGR) or 10 (normal growth) at birth and exposed to room air or 75% oxygen for 14 days. Half of the pups were treated with the TLR4 inhibitor TAK-242 from birth or beginning at day 3. After 14 days, pulmonary arterial pressure was evaluated by echocardiography and hearts were examined for right ventricular hypertrophy (RVH). Lungs and serum samples were analyzed by western blotting and immunohistochemistry.

**Results:**

Postnatal growth restriction + hyperoxia increased pulmonary arterial pressure and RVH with trends toward increased plasma IL1β and decreased IκBα, the inhibitor of NFκB, in lung tissue. Treatment with the TLR4 inhibitor attenuated PH and inflammation.

**Conclusion:**

Postnatal growth restriction induces an increase in intestinal Enterobacteriaceae leading to PH. Activation of the TLR4 pathway is a promising mechanism by which intestinal dysbiosis impacts the developing lung.

## Introduction

Pulmonary hypertension (PH) is an increase in pulmonary vascular resistance resulting in a decrease in pulmonary blood flow and right ventricular hypertrophy (RVH). The incidence of PH among extremely premature infants (birth weight less than 1000 g) is as high as 18% and increases to 25–40% among premature infants with bronchopulmonary dysplasia (BPD), a chronic lung disease (1, 2). In this population, PH is associated with very high morbidity and 50% mortality (3). PH is often not diagnosed until the patient develops severe right ventricular dysfunction. Current screening methods are unreliable, and no early biomarkers of PH exist.

A large cohort study found that 79% of premature infants with gestational age <27 weeks displayed poor growth after birth (post-natal growth restriction, PNGR) (4). PNGR is associated with a sustained elevation in C-reactive protein (5) and increases the risk of PH, BPD and other diseases of prematurity including necrotizing enterocolitis (NEC), an inflammatory disease of the intestines (6–8). In a recent prospective study of PH, among extremely preterm infants with BPD (mean gestational age at birth 26 weeks) evaluated at 36 to 38 weeks corrected gestational age, 13/44 (30%) patients with PH had a history of NEC, while only 8/115 (7%) patients without PH had a history of NEC [adjusted odds ratio 5.5 (95% confidence intervals 1.9, 15.4)] (9), suggesting an association between inflammation in the gut and pulmonary vascular disease. A meta-analysis confirmed a strong association between NEC and PH particularly among infants with BPD (RR 3.4 with 95% confidence intervals 1.1 and 10.2) (10).

Similar to preterm infants, rats are born in the saccular stage of lung development. Neonatal rats exposed to hyperoxia (75–95% O_2_) for 14 days develop PH, RVH, pulmonary vascular remodeling, and alveolar simplification characteristic of preterm infants with BPD (11). We have shown in a novel rodent model that PNGR, achieved by increasing litter size from 10 to 17 pups, triggers PH and amplifies the adverse effects of hyperoxia at 2 weeks of age (12, 13). This age is roughly equivalent to a human infant at 6–12 months (14), a common time of death for premature infants with PH.

Associations between nutrition, the intestinal microbiota and immune responses in distant sites such as the lung, brain and liver have prompted study of the gut-lung, gut-brain, and gut-liver axes. We recently reported that PNGR, but not hyperoxia, alters the intestinal microbiota in rats at 14 days (15). Partially correcting the dysbiosis with a probiotic strain of *Lactobacillus reuteri* attenuates PNGR-induced PH (15). We also reported an increase in pro-inflammatory Gram-negative Enterobacteriaceae in the distal small bowel of rat pups exposed to both PNGR and hyperoxia (15). Recognition of lipopolysaccharide (LPS) in the cell wall of Enterobacteriaceae by the host Toll-like receptor (TLR)4 is important in the pathogenesis of NEC, and both inhibition of TLR4 and manipulation of the intestinal microbiota with probiotic organisms prevents this disease (16, 17). TLR4 signaling is also important in lung injury and inflammation (18). In a rodent model of NEC, lung injury is prominent and can be attenuated by deletion of TLR4 from the pulmonary epithelium (19). Furthermore, activation of TLR4 in the NEC model induced expression of chemokine ligand 25 (CC25) resulting in recruitment of Th17 cells to the lungs (20). From these studies we hypothesized that TLR4 –induced inflammation in the intestines is an important mechanism by which PNGR-associated dysbiosis triggers PH in rat pups. The goal of this study was to investigate the role of TLR4 in the developing gut-lung axis.

## Materials and Methods

### Animals

The animal protocol was approved by the Institutional Animal Care and Use Committee at UC Davis. Timed-pregnant Sprague Dawley dams at E14-E16 were ordered from Charles River Laboratories (Wilmington, MA, United States). Rats were housed in plastic cages with a 12 h dark:light cycle and allowed to feed *ad libitum* with a standard diet (2018 Teklad from Harlan). After birth, pups were pooled and randomly assigned to litters of 10 pups (normal litter size, N) or 17 pups (restricted litter size, R). Additionally, pups were randomly assigned to cages maintained in room air (A) or exposed to 75% oxygen (O) in a plexiglass chamber (Biospherix, Lacona, NY, United States) continuously, and dams were rotated with the appropriate control or PNGR dam every 24 h. As we have shown previously, the pups tolerate hyperoxia for 14 days without mortality (12). Some pups in each group were injected subcutaneously with the TLR4 inhibitor TAK-242 (Cayman Chemicals, Ann Arbor, MI, United States) (Resatorvid, 3 mg/kg/day from birth) or with vehicle alone (5% ethanol). The dose was chosen based on a previous study in a mouse sepsis model (21). At postnatal day 14, the pups were analyzed by echocardiography, weighed and euthanized for tissue harvest. Pups were euthanized by exposure to CO_2_ followed by cardiac puncture and exsanguination, and plasma was collected by centrifugation in heparin-treated tubes (Thermo Fisher Scientific) and stored at −80°C. Hearts and lungs, were snap-frozen in liquid nitrogen and stored at −80°C. The intestinal microbiota was not evaluated for this series of experiments, but has previously been reported for this model (15).

### Echocardiography

At day 14, echocardiography was performed on pups under light anesthesia with isoflurane using a VisualSonics VIVO 2100 *in vivo* ultrasound imaging system (VisualSonics, Toronto, ON, Canada) to determine the ratio of the pulmonary acceleration time (PAT) to the total ejection time (ET) a marker of PH as previously described (12).

### Measurement of Right Ventricular Hypertrophy (RVH)

Fulton’s index [the weight of the right ventricle (RV) divided by the weight of the left ventricle (LV) + septum] was determined to assess RVH. Additionally, RV and LV + septum weights were normalized to body weight (22).

*Plasma IL-1b* was quantified using the Rat IL-1 beta Platinum ELISA kit (Thermo Fisher Scientific, Waltham, MA, United States) according to the manufacturer’s instructions.

*Western blots* were performed on lung tissue as previously described (12). Briefly, lung tissue was suspended in RIPA buffer containing protease and phosphatase inhibitors and sonicated on ice. Protein content was determined by the Bradford method and Western blotting performed using 1:500 dilution of mouse anti-IκB-α antibody (sc-1643, Santa Cruz Biotechnology, Dallas, TX, United States) at 4°C overnight followed by a 60 min incubation with an anti-mouse secondary antibody conjugated to horseradish peroxidase (Santa Cruz). Blots were then probed for β-actin (ab6276, 1:4000, Abcam, Cambridge, MA, United States) for 60 min at room temperature. Chemiluminescence generated by Super Signal West Femto substrate (Thermo Fisher Scientific) was detected and quantified using a Kodak Image Station and software. Signals were normalized to β-actin and expressed as fold change relative to OR animals.

### Statistical Analysis

Data are presented as means ± SEM. “N” represents the number of animals in each group. Groups were compared with one-way ANOVA (Stata 12.1, College Station, TX, United States). If the *F* test was significant, a Scheffe *post hoc* test was performed. The independent variables were considered significant at *p* < 0.05.

## Results

**FIGURE 1:**
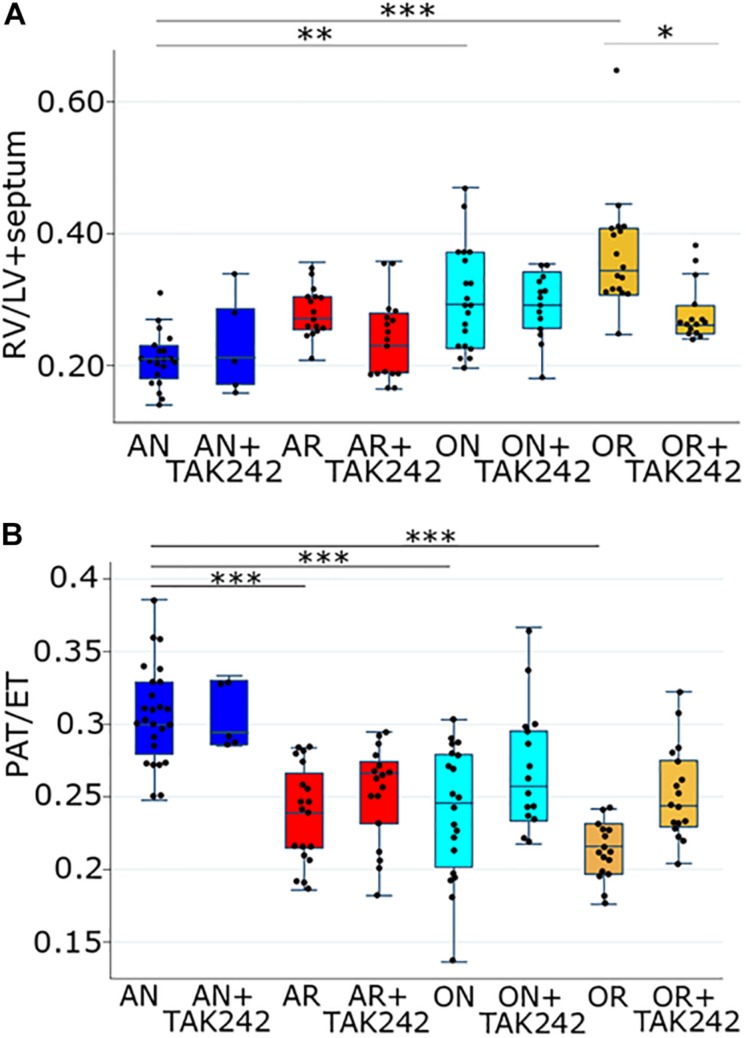
**(A)** Fulton’s index (right ventricular weight divided by the combined weight of the left ventricle and the intraventricular septum) in the four groups of the PNGR model with and without treatment with the TLR4 inhibitor TAK-242. One way ANOVA *p* < 0.001, AN vs. AR *p* = 0.2, AN vs. ON *p* = 0.01, AN vs. OR *p* < 0.001, OR vs. OR + TAK242 *p* = 0.04 (Scheffe). Comparing just the AN vs. AR (not considering the other groups), using a *t*-test, *p* < 0.001. Number of animals AN = 21, AN + TAK242 = 5, AR = 16, AR + TAK242 = 17, ON = 20, ON + TAK242 = 14, OR = 17, OR + TAK242 = 15. **(B)** Ratio of PAT to total ejection time (PAT/ET) in the four groups of the PNGR model with and without treatment with the TLR4 inhibitor TAK-242. One way ANOVA *p* < 0.001, AN vs. AR *p* < 0.001, AN vs. ON *p* < 0.001, AN vs. OR *p* < 0.001, OR vs. OR + TAK242 *p* = 0.18 (Scheffe). Comparing just the OR vs. OR + TAK242 (not considering the other groups), using a *t*-test, *p* < 0.001. Number of animals AN = 25, AN + TAK242 = 5, AR = 19, ARTAK242 = 17, ON = 20, ON + TAK242 = 15, OR = 16, OR + TAK242 = 17. Asterisks in both A and B are from the Scheffe post hoc test: ^∗^ *p* < 0.05, ^∗∗^ *p* < 0.01, ^∗∗∗^ *p* < 0.001. AN = room air, normal litter size; AR = room air, growth-restricted litter size; ON = hyperoxia, normal litter size; OR = hyperoxia, growth-restricted litter size.

Our previous study identified an increase in Enterobacteriaceae in the distal small bowel of rat pups exposed to PNGR and hyperoxia (15). To determine if activation of TLR4 by Enterobacteriaceae is involved in the development of PH in these rats, we first determined the efficacy of the TLR4 antagonist TAK-242 to attenuate PH. Increased pulmonary artery pressure results in RVH. As we have shown previously (12), PNGR and hyperoxia alone increase Fulton’s index (the ratio of RV weight to LV + septum weight) with a further increase in Fulton’s index when both are combined (Figure 1A). Daily treatment with TAK-242 attenuated RVH in pups exposed to PNGR with and without hyperoxia, but not in pups exposed to hyperoxia alone (Figure 1A).

The ratio of the PAT to total ejection time (PAT/ET) detected by echocardiography decreases with increased pulmonary artery pressures. As we have shown previously (12), PAT/ET ratios were significantly decreased in pups exposed to PNGR or hyperoxia alone, and were decreased further in pups exposed to both (Figure 1B). Daily treatment with TAK-242 attenuated the decrease in PAT/ET ratios in pups exposed to hyperoxia with PNGR, but not in pups exposed to PNGR or hyperoxia alone (Figure 1B).

**FIGURE 2:**
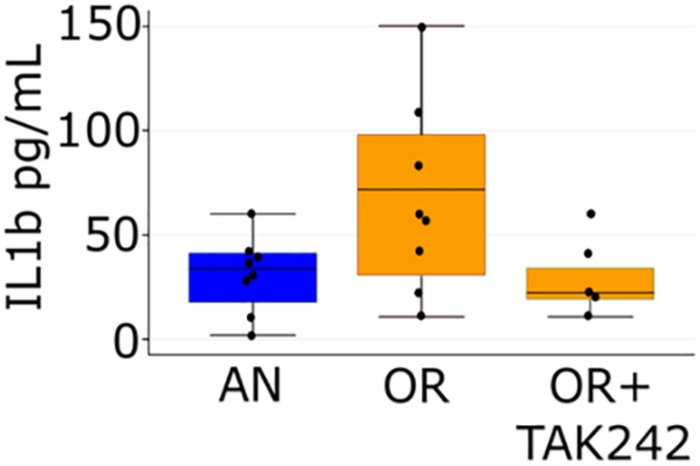
Plasma IL-1β measured by ELISA at day 14 in pups from the air, normal litter size group and the hyperoxia, PNGR group with and without treatment with the TLR4 inhibitor TAK-242. One way ANOVA *p* = 0.046, AN vs. OR *p* = 0.08 and OR vs. OR + TAK242 *p* = 0.12 (Scheffe). Number of animals: AN = 8, OR = 8, OR + TAK242 = 5.

We opted to focus the remaining experiments on the PNGR and hyperoxia group for four reasons: (1) we have previously demonstrated that intestinal dysbiosis is most severe in the PNGR and hyperoxia group with the largest increases in Enterobacteriaceae, (2) this group consistently has the most severe phenotype in our model, (3) this group had a significant attenuation of both RVH and PAT/ET ratio, with TLR4 inhibition and (4) this group most closely reflects extremely premature infants at the highest risk for PH (those with BPD and poor postnatal growth). We next looked at circulating levels of the cytokine IL-1β, a downstream component of TLR4-induced inflammatory responses. A strong trend toward higher plasma levels was seen in pups exposed to PNGR and hyperoxia relative to controls, while daily treatment with TAK-242 trended toward decreased circulating IL-1β (Figure 2).

**FIGURE 3:**
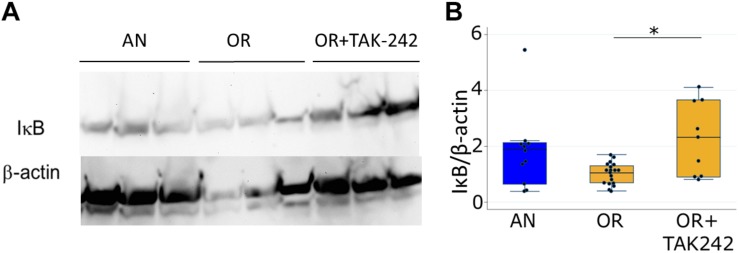
Lung IκBα measured by western blotting at 14 days in pups from the air, normal litter size group and the hyperoxia, PNGR group with and without treatment with the TLR4 inhibitor TAK-242. **(A)** Representative blot **(B)**. Each band was normalized to β-actin and to OR (each point represents a fold change compared to the mean OR value) One way ANOVA *p* < 0.01, AN vs. OR *p* = 0.11 and OR vs. OR + TAK-242 *p* < 0.02 (^∗^Scheffe). Number of animals: AN = 11, OR = 19, OR + TAK242 = 9.

We quantified levels of IκBα protein in lung as a marker of lung inflammation. IκBα is an inhibitory protein of the key pro-inflammatory transcription factor NFκB, and decreases in IκBα indicate an increase in NFκB-mediated inflammation. IκBα protein trended to a decrease in lungs from rats exposed to PNGR and hyperoxia relative to controls, while daily treatment with TAK-242 significantly increased IκBα levels (Figure 3).

**FIGURE 4:**
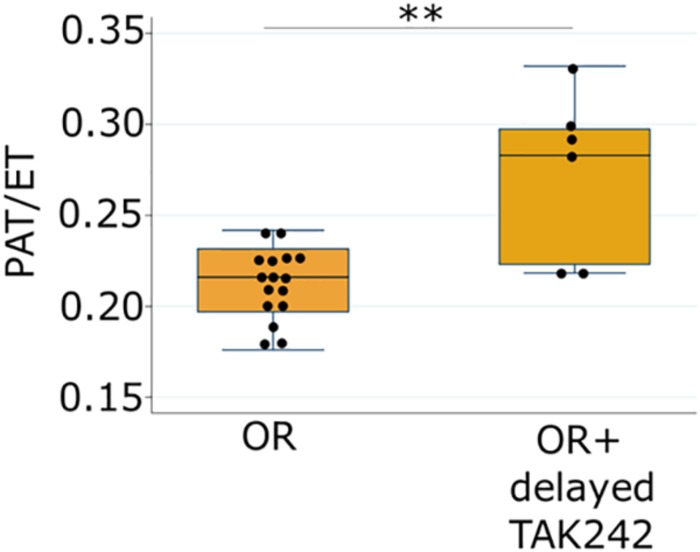
Ratio of pulmonary acceleration time to total ejection time (PAT/ET) in pups exposed to hyperoxia and PNGR model with and without delayed treatment (day 3) with the TLR4 inhibitor TAK-242. ^∗∗^*p* < 0.01. Number of animals: OR = 16, OR + delayed TAK242 = 6.

In our proposed pathway, intestinal dysbiosis precedes and initiates TLR4 signaling. This raises the possibility that delayed treatment, either to alter the intestinal microbiota or to inhibit TLR4 targeting may be effective in attenuating PH, an advantage in the management of a disease that is not apparent in the premature infant in the first days and weeks of life. To test this hypothesis, we performed additional experiments in which the pups were divided into the four groups on day 1 as usual, but the intervention was not begun until day of life 3. Delaying treatment with TAK-242 until postnatal day 3 still led to significantly increased PAT/ET on day 14 in pups exposed to PNGR and hyperoxia (Figure 4) indicating attenuated PH.

## Discussion

Postnatal growth restriction is common with very premature birth and increases the risk of BPD and PH. Retrospective cohort studies have demonstrated associations between decreased caloric intake in the first weeks of life and BPD in very preterm infants (6, 7, 23), and limited studies of aggressive nutrition in the first weeks of life have shown benefit in decreasing BPD in this same population (24). NEC is also a risk factor for PH in very preterm infants both with and without BPD (9), supporting the hypothesis that inflammation in the gut impacts the developing lung vasculature.

**FIGURE 5:**
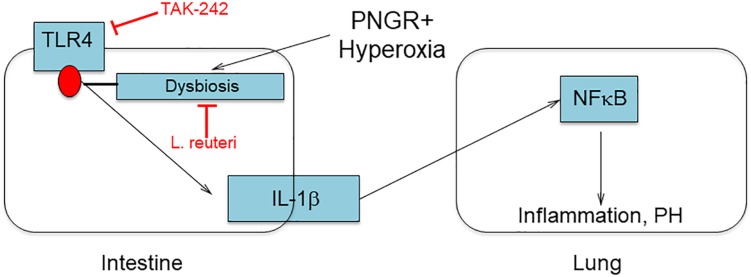
Hypothesized role of dysbiosis and TLR4 signaling in the developing gut-lung axis.

Many rodent models of PH involve exposing neonatal pups to hyperoxia, although our model including a component of PNGR more closely approximates clinical conditions in extremely premature infants. As such it is a powerful tool to investigate the underlying mechanisms whereby the most vulnerable extremely low birth weight premature infants (those with poor growth receiving supplemental oxygen) are at greatest risk of developing cardiovascular diseases. From these data and our previously published study (15) we hypothesized that PNGR combined with hyperoxia triggers intestinal dysbiosis including elevated Enterobacteriaceae. Blooms of Enterobacteriaceae have been identified just prior to the onset of necrotizing enterocolitis in premature infants (25) and are a signature of dysbiosis in many disease processes (26). We further hypothesized that the resultant activation of TLR4 by Enterobacteriaceae in the intestines triggers an inflammatory response including elevated circulating IL-1β. This transduces the inflammatory signal to the lungs activating NFκB, leading to PH and RVH (Figure 5). The present study identifying a role for TLR4 signaling in PH induced by PNGR and hyperoxia supports this hypothesis. We previously demonstrated that the probiotic *L. reuteri* DSM 17938 reverses dysbiosis and attenuates PH and RVH (15), and inhibiting TLR4 signaling likewise attenuates PH and RVH as we show here.

Furthermore, our data suggest that delayed targeting of TLR4 signaling is still effective in attenuating PH. We do not yet know the windows of benefit for either probiotic administration or TLR4 inhibition for successful attenuation of PH in our model, but a potential treatment strategy for preterm infants may involve early probiotic treatment with subsequent targeting of TLR4 signaling in high risk infants.

Studies investigating the gut-lung axis have prompted the hypothesis that intestinal dysbiosis is an important driver of systemic inflammation (27). These associations are particularly important in preterm neonates with immature gut and lung immune responses. Studies of human milk are particularly relevant in this population. Human milk feeding decreases the risk of NEC (28). Meta-analyses suggest a benefit in feeding mother’s own milk (29) and pasteurized donor human milk (30) in the prevention of BPD. In organoids derived from the terminal ileum of mouse pups, human milk exosomes have been shown to attenuate LPS induced activation of TLR4 (31). Human milk oligosaccharides interact with TLR4 on the surface of dendritic cells inducing immune tolerance through increased generation of regulatory T cells and attenuation of LPS-induced expression of IL6 and TNFα (32). It is also possible that TLR4 is important in maintenance of stem cells in the developing gut and lung. In the developing intestinal tract, intestinal stem cells express TLR4 which regulates proliferation and apoptosis (33). In a lung injury model, deletion of TLR4 impairs the renewal capacity of lung stem cells (34). Conversely, in a model of neonatal PH triggered by intra-amniotic injection of PBS, human mesenchymal stem cells decrease expression of TLR4, NFκB, and TNFα in the heart and attenuate PH (35).

Toll-like receptors are important in recognition of pathogen-associated molecular patterns and triggering of innate immune responses in both the gut and the lung, as demonstrated in the studies of TLR4 in NEC-associated lung injury noted in the introduction. TLR4 activates IL-1β transcription via NFκB (36). The role of TLR4 in regulating pulmonary vasculogenesis has also been explored. Adult TLR4-deficient mice do not develop PH when exposed to hypoxia (37). Stimulation of TLR4 on platelets leads to platelet activation and aggregation exacerbating PH (and as a result selective knockout of TLR4 on platelets is protective) (38). The increase in Enterobacteriaceae in our PNGR model suggests a potential role for TLR4 in the intestine and/or the lung in the resultant PH. Probiotic microbes impact host immune responses including downregulation of TLR4 (39), chemokines and cytokines (40–46), suppression of T-helper 2 responses (47) decrease in intestinal permeability (48–51), alteration of intestinal motility (52, 53), and production of short chain fatty acids (54, 55).

Lung inflammation is involved in the development of PH in humans and animal models (56). We believe the current study is the first to demonstrate a potential role for an inflammatory response initiating in the intestines in PNGR/hyperoxia-induced PH. Our studies do not rule out the possibility of a TLR4 response induced in the lung following simultaneous exposure to PNGR and hyperoxia; direct measurement of TLR4 in both the gut and lung would be valuable to address this possibility. Activation of lung TLR4 using aerosolized LPS results in elevated IL-1β in bronchiolar lavage fluid in mice (57), while treatment with the anti-inflammatory molecule dioscin suppresses various pro-inflammatory molecules including TLR4, IL-1β and NFκB in the lungs of rats injected with LPS (58). Our current study indicated that subcutaneous TAK-242 is effective at attenuating PH in our model. Further studies comparing the efficacy of TAK-242 delivered intranasally or via gavage may identify where TLR4 is activated in rats exposed to PNGR and hyperoxia. Measurement of IL1β in both intestinal and lung tissue in this model would be of value in future studies.

In the current study we demonstrate that IκBα levels were decreased in the lungs of rats exposed to PNGR and hyperoxia, while TAK-242 prevented this decrease. Increased NFκB activity is evident in explanted lungs of patients with idiopathic PH (59), and an NFκB decoy delivery into lungs prevents monocrotaline-induced NFκB activity and PH in rats (59) suggesting that elevated lung NFκB activity plays a central role in the pathogenesis of PH. We have previously shown a decrease in IκB in the lungs and pulmonary arteries in a lamb model of persistent PH of the newborn suggesting a potential role for NFκB-target genes in pulmonary vascular remodeling (60). Direct measurement of NFκB in lung tissue in this model would be of value.

In summary, we show that TLR4 inhibition attenuated PH, RVH, and decreased lung IκBα activity in rat pups exposed to PNGR and hyperoxia with a trend toward decreasing elevated circulating IL-1β. Further elucidation of the underlying mechanisms may identify crucial spatial (intestinal and pulmonary) and temporal targets to improve clinical outcomes of low birth weight preterm infants at risk of developing PH.

## Data Availability Statement

The datasets generated for this study are available on request to the corresponding author.

## Ethics Statement

The animal study was reviewed and approved by the UC Davis Institutional Animal Care and Use Committee.

## Author Contributions

SW contributed to study design and data analysis and wrote initial draft. KG, KH, SM, and AH performed the analyses. CW and PT performed the animal experiments. RS, SL, NC, and MU contributed to study design and data analysis. All authors approved the final manuscript.

## Conflict of Interest

The authors declare that the research was conducted in the absence of any commercial or financial relationships that could be construed as a potential conflict of interest.
